# 2,6,6-Trimethyl­cyclo­hex-2-ene­carb­oxy­lic acid

**DOI:** 10.1107/S1600536812002668

**Published:** 2012-01-31

**Authors:** Rajasekaran Parthasarathy, Samson Jegan Jenniefer, Packianathan Thomas Muthiah, Nagarajan Sulochana

**Affiliations:** a Department of Chemistry, National Institute of Technology, Tiruchirappalli 620 015, India; b School of Chemistry, Bharathidasan University, Tiruchirappalli 620 024, Tamil Nadu, India; c Department of Chemistry, National Institute of Technology, Karaikal 609 605, India

## Abstract

In the title crystal structure, C_10_H_16_O_2_, inversion-related mol­ecules are linked by pairs of O—H⋯O hydrogen bonds involving carboxyl groups to form *R*_2_^2^(8) dimers. The cyclo­hexene ring displays a half-chair conformation.

## Related literature

For information on the title compound as used as a key inter­mediate in chemical synthesis, see: Eugster *et al.* (1969 ▶); Naef & Decorzant (1986 ▶); Snowden *et al.* (1982 ▶); Fehr & Galindo (1986 ▶, 1995 ▶); Heather *et al.* (1976 ▶). For hydrogen-bond graph-set notation, see: Etter *et al.* (1990 ▶); Bernstein *et al.* (1995 ▶).
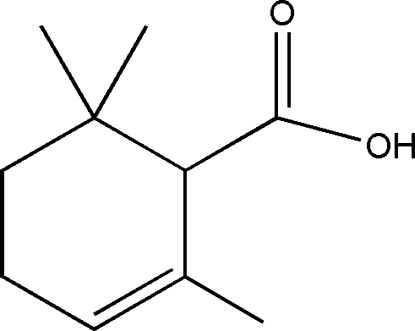


## Experimental

### 

#### Crystal data

C_10_H_16_O_2_
*M* *_r_* = 168.23
Monoclinic, 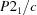

*a* = 7.6817 (1) Å
*b* = 10.4137 (2) Å
*c* = 13.4421 (2) Å
β = 112.924 (1)°
*V* = 990.38 (3) Å^3^
*Z* = 4
Mo *K*α radiation
μ = 0.08 mm^−1^
*T* = 296 K
0.09 × 0.08 × 0.05 mm

#### Data collection

Bruker SMART APEXII CCD diffractometer
Absorption correction: multi-scan (*SADABS*; Bruker, 2008 ▶) *T*_min_ = 0.993, *T*_max_ = 0.996
10045 measured reflections
2158 independent reflections
1560 reflections with *I* > 2σ(*I*)
*R*_int_ = 0.026

#### Refinement

*R*[*F* ^2^ > 2σ(*F* ^2^)] = 0.049
*wR*(*F* ^2^) = 0.134
*S* = 1.03
2158 reflections
120 parameters
H atoms treated by a mixture of independent and constrained refinement
Δρ_max_ = 0.25 e Å^−3^
Δρ_min_ = −0.17 e Å^−3^

### 

Data collection: *APEX2* (Bruker, 2008 ▶); cell refinement: *SAINT* (Bruker, 2008 ▶); data reduction: *SAINT*; program(s) used to solve structure: *SHELXS97* (Sheldrick, 2008 ▶); program(s) used to refine structure: *SHELXL97* (Sheldrick, 2008 ▶); molecular graphics: *PLATON* (Spek, 2009 ▶); software used to prepare material for publication: *PLATON*.

## Supplementary Material

Crystal structure: contains datablock(s) global, I. DOI: 10.1107/S1600536812002668/lh5406sup1.cif

Structure factors: contains datablock(s) I. DOI: 10.1107/S1600536812002668/lh5406Isup2.hkl

Supplementary material file. DOI: 10.1107/S1600536812002668/lh5406Isup3.cml

Additional supplementary materials:  crystallographic information; 3D view; checkCIF report

## Figures and Tables

**Table 1:** Hydrogen-bond geometry (Å, °)

*D*—H⋯*A*	*D*—H	H⋯*A*	*D*⋯*A*	*D*—H⋯*A*
O2—H2*A*⋯O1^i^	1.01 (4)	1.64 (4)	2.646 (2)	178 (4)

Symmetry code: (i) 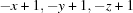.

**Fig. 1.**
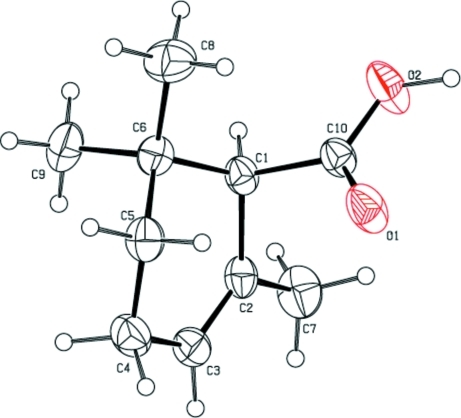
The molecular structure of the title compound, with anisotropic displacement ellipsoids drawn at 50% probability level.

**Fig. 2.**
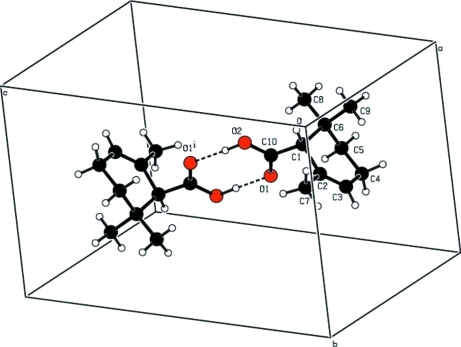
A centrosymmetric R22(8) hydrogen-bonded dimer unit, with hydrogen bonds shown as dashed lines. For symmetry code (i), see Table 1.

## supplementary crystallographic information

### Comment

The title compound is a key intermediate for the synthesis of aroma compounds such as alpha, beta methyl *cyclo* geranate (Eugster *et al.*, 1969), alpha damascone (Naef & Decorzant, 1986; Snowden *et al.*, 1982), beta damascone (Fehr & Galindo, 1986), gamma damascone (Fehr & Galindo, 1995) and strigol which is a highly potent stimulant for the germination of seeds of parasitic weeds striga and orobanche (Heather *et al.*, 1976). Moreover, the 2,6,6-tri methyl*cyclo*hexenyl moiety is a basic moiety for natural product of carotenoid, which is a naturally occurring organic pigment in the chloroplasts and chromoplasts of plants. Herein, we report the crystal and molecular structure of the title compound (Fig. 1). In the crystal, inversion-related molecules are connected via a pair of O—H···O hydrogen bonds, (Table 1) forming a cyclic dimer [graph-set *R*^2^_2_(8) (Etter *et al.*, 1990; Bernstein *et al.*, 1995)] (Fig. 2). This type of cyclic donor···acceptor···acceptor···donor interaction involving O—H···O hydrogen bonds is frequently observed in carboxylic acids

### Experimental

A solution of 8 g (0.07 mol) of 80% sodium chlorite in 70 ml H_2_O was added drop wise for 2 h at room temperature to a stirred mixture of 6.6 g (0.05 mol) of *cyclo* citral in 50 ml Me_2_SO and of 1.6 g NaH_2_PO_4_ in 20 ml of water. The mixture was left overnight at room temperature, then 100 ml of water was added to the reaction mixture. The solid geranic acid was collected and crystallized from hexane.

### Refinement

The H atom attached to O2 was located in a difference Fourier map and refined freely. The remaining H atoms were placed in geometrically idealized positions and constrained to ride on their parent atoms, with C—H distances in the range 0.93–0.97 Å, and with *U*_iso_(H) set at 1.2*U*_eq_(C), except for the methyl hydrogen atoms which were refined with *U*_iso_(H) set at 1.5*U*_eq_(C).

### Crystal data

**Table:**

C_10_H_16_O_2_	*F*(000) = 368
*M**_r_* = 168.23	*D*_x_ = 1.128 Mg m^−^^3^
Monoclinic, *P*2_1_/*c*	Mo *K*α radiation, λ = 0.71073 Å
Hall symbol: -P 2ybc	Cell parameters from 3232 reflections
*a* = 7.6817 (1) Å	θ = 2.9–25.1°
*b* = 10.4137 (2) Å	µ = 0.08 mm^−^^1^
*c* = 13.4421 (2) Å	*T* = 296 K
β = 112.924 (1)°	Prism, colourless
*V* = 990.38 (3) Å^3^	0.09 × 0.08 × 0.05 mm
*Z* = 4	

### Data collection

**Table:**

Bruker SMART APEXII CCD diffractometer	2158 independent reflections
Radiation source: fine-focus sealed tube	1560 reflections with *I* > 2σ(*I*)
graphite	*R*_int_ = 0.026
φ and ω scans	θ_max_ = 27.0°, θ_min_ = 2.6°
Absorption correction: multi-scan (*SADABS*; Bruker, 2008)	*h* = −9→8
*T*_min_ = 0.993, *T*_max_ = 0.996	*k* = −13→8
10045 measured reflections	*l* = −17→17

### Refinement

**Table:**

Refinement on *F*^2^	Primary atom site location: structure-invariant direct methods
Least-squares matrix: full	Secondary atom site location: difference Fourier map
*R*[*F*^2^ > 2σ(*F*^2^)] = 0.049	Hydrogen site location: inferred from neighbouring sites
*wR*(*F*^2^) = 0.134	H atoms treated by a mixture of independent and constrained refinement
*S* = 1.03	*w* = 1/[σ^2^(*F*_o_^2^) + (0.0541*P*)^2^ + 0.2979*P*] where *P* = (*F*_o_^2^ + 2*F*_c_^2^)/3
2158 reflections	(Δ/σ)_max_ = 0.003
120 parameters	Δρ_max_ = 0.25 e Å^−^^3^
0 restraints	Δρ_min_ = −0.17 e Å^−^^3^

### Special details

**Table:**

Geometry. Bond distances, angles *etc*. have been calculated using the rounded fractional coordinates. All su's are estimated from the variances of the (full) variance-covariance matrix. The cell e.s.d.'s are taken into account in the estimation of distances, angles and torsion angles
Refinement. Refinement on *F*^2^ for ALL reflections except those flagged by the user for potential systematic errors. Weighted *R*-factors *wR* and all goodnesses of fit *S* are based on *F*^2^, conventional *R*-factors *R* are based on *F*, with *F* set to zero for negative *F*^2^. The observed criterion of *F*^2^ > σ(*F*^2^) is used only for calculating -*R*-factor-obs *etc*. and is not relevant to the choice of reflections for refinement. *R*-factors based on *F*^2^ are statistically about twice as large as those based on *F*, and *R*-factors based on ALL data will be even larger.

### Fractional atomic coordinates and isotropic or equivalent isotropic displacement parameters (Å^2^)

**Table:**

	*x*	*y*	*z*	*U*_iso_*/*U*_eq_	
O1	0.5749 (2)	0.54166 (13)	0.40375 (11)	0.0729 (6)	
O2	0.4766 (2)	0.35104 (14)	0.43135 (12)	0.0793 (6)	
C1	0.5737 (2)	0.36939 (16)	0.28421 (12)	0.0432 (5)	
C2	0.4543 (2)	0.44209 (16)	0.18282 (12)	0.0464 (5)	
C3	0.5312 (3)	0.52452 (17)	0.13678 (13)	0.0531 (6)	
C4	0.7364 (3)	0.5534 (2)	0.17489 (16)	0.0612 (7)	
C5	0.8506 (2)	0.49910 (18)	0.28571 (15)	0.0548 (6)	
C6	0.7857 (2)	0.36479 (17)	0.30219 (13)	0.0495 (5)	
C7	0.2461 (3)	0.4137 (2)	0.13917 (17)	0.0723 (8)	
C8	0.9058 (3)	0.3161 (2)	0.41584 (17)	0.0770 (8)	
C9	0.8045 (3)	0.2706 (2)	0.21865 (18)	0.0676 (7)	
C10	0.5421 (2)	0.42734 (16)	0.37950 (12)	0.0467 (5)	
H1	0.527 (2)	0.2818 (16)	0.2775 (12)	0.042 (4)*	
H2A	0.459 (5)	0.394 (4)	0.494 (3)	0.167 (13)*	
H3	0.45030	0.56780	0.07570	0.0640*	
H4A	0.75350	0.64580	0.17680	0.0730*	
H4B	0.78430	0.51850	0.12360	0.0730*	
H5A	0.84030	0.55630	0.34010	0.0660*	
H5B	0.98260	0.49550	0.29580	0.0660*	
H7A	0.19700	0.43390	0.19300	0.1080*	
H7B	0.22580	0.32430	0.12080	0.1080*	
H7C	0.18260	0.46470	0.07580	0.1080*	
H8A	0.85880	0.23430	0.42710	0.1160*	
H8B	0.89920	0.37630	0.46830	0.1160*	
H8C	1.03480	0.30720	0.42340	0.1160*	
H9A	0.72010	0.29590	0.14750	0.1010*	
H9B	0.77290	0.18550	0.23350	0.1010*	
H9C	0.93220	0.27160	0.22290	0.1010*	

### Atomic displacement parameters (Å^2^)

**Table:**

	*U*^11^	*U*^22^	*U*^33^	*U*^12^	*U*^13^	*U*^23^
O1	0.1202 (12)	0.0524 (8)	0.0696 (9)	−0.0172 (8)	0.0626 (9)	−0.0177 (6)
O2	0.1320 (14)	0.0630 (9)	0.0742 (9)	−0.0294 (8)	0.0741 (10)	−0.0186 (7)
C1	0.0504 (9)	0.0410 (9)	0.0432 (8)	−0.0068 (7)	0.0238 (7)	−0.0081 (6)
C2	0.0455 (9)	0.0551 (10)	0.0408 (8)	−0.0020 (7)	0.0193 (7)	−0.0111 (7)
C3	0.0575 (10)	0.0604 (11)	0.0418 (8)	0.0047 (8)	0.0199 (8)	0.0005 (7)
C4	0.0651 (12)	0.0624 (12)	0.0653 (11)	−0.0064 (9)	0.0354 (10)	0.0043 (9)
C5	0.0451 (9)	0.0597 (11)	0.0611 (10)	−0.0083 (8)	0.0222 (8)	−0.0074 (8)
C6	0.0454 (9)	0.0515 (10)	0.0516 (9)	0.0014 (7)	0.0190 (7)	−0.0027 (7)
C7	0.0501 (11)	0.0970 (16)	0.0650 (12)	−0.0081 (10)	0.0172 (9)	−0.0101 (11)
C8	0.0661 (13)	0.0828 (15)	0.0682 (12)	0.0081 (11)	0.0110 (10)	0.0130 (11)
C9	0.0648 (12)	0.0620 (12)	0.0864 (14)	0.0074 (9)	0.0408 (11)	−0.0119 (10)
C10	0.0563 (10)	0.0460 (10)	0.0424 (8)	−0.0067 (8)	0.0244 (7)	−0.0056 (7)

### Geometric parameters (Å, °)

**Table:**

O1—C10	1.234 (2)	C3—H3	0.9300
O2—C10	1.282 (2)	C4—H4A	0.9700
O2—H2A	1.01 (4)	C4—H4B	0.9700
C1—C6	1.551 (2)	C5—H5A	0.9700
C1—C10	1.519 (2)	C5—H5B	0.9700
C1—C2	1.516 (2)	C7—H7A	0.9600
C2—C7	1.503 (3)	C7—H7B	0.9600
C2—C3	1.324 (3)	C7—H7C	0.9600
C3—C4	1.486 (3)	C8—H8A	0.9600
C4—C5	1.513 (3)	C8—H8B	0.9600
C5—C6	1.530 (3)	C8—H8C	0.9600
C6—C8	1.531 (3)	C9—H9A	0.9600
C6—C9	1.540 (3)	C9—H9B	0.9600
C1—H1	0.971 (17)	C9—H9C	0.9600
			
O1···C5	3.133 (2)	H2A···O1^i^	1.64 (4)
O1···C8	3.418 (3)	H2A···O2^i^	2.81 (4)
O1···C10^i^	3.382 (2)	H2A···C10^i^	2.52 (4)
O1···O2^i^	2.646 (2)	H2A···H2A^i^	2.28 (6)
O2···O1^i^	2.646 (2)	H3···H7C	2.3200
O2···C8	3.403 (3)	H3···C3^iii^	3.0700
O1···H8B	2.8700	H4A···O2^iv^	2.7900
O1···H2A^i^	1.64 (4)	H4B···C9	2.8600
O1···H5A	2.5000	H4B···H9A	2.4200
O2···H4A^ii^	2.7900	H5A···O1	2.5000
O2···H2A^i^	2.81 (4)	H5A···C10	2.8800
C3···C9	3.288 (3)	H5A···H8B	2.4700
C3···C3^iii^	3.555 (2)	H5B···H8C	2.5300
C5···O1	3.133 (2)	H5B···H9C	2.5000
C8···O2	3.403 (3)	H7A···C10	2.8500
C8···O1	3.418 (3)	H7B···H1	2.4900
C9···C3	3.288 (3)	H7C···H3	2.3200
C10···O1^i^	3.382 (2)	H8A···C10	3.0300
C2···H9A	2.7300	H8A···H9B	2.4800
C3···H3^iii^	3.0700	H8B···O1	2.8700
C3···H1^iv^	3.018 (17)	H8B···C10	2.5800
C3···H9A	2.7600	H8B···H5A	2.4700
C4···H9A	2.7000	H8C···H5B	2.5300
C9···H4B	2.8600	H8C···H9C	2.5200
C10···H5A	2.8800	H9A···C2	2.7300
C10···H8A	3.0300	H9A···C3	2.7600
C10···H8B	2.5800	H9A···C4	2.7000
C10···H7A	2.8500	H9A···H4B	2.4200
C10···H2A^i^	2.52 (4)	H9B···H1	2.4100
H1···H7B	2.4900	H9B···H8A	2.4800
H1···H9B	2.4100	H9C···H5B	2.5000
H1···C3^ii^	3.018 (17)	H9C···H8C	2.5200
			
C10—O2—H2A	113 (2)	C5—C4—H4A	109.00
C2—C1—C10	108.68 (13)	C5—C4—H4B	109.00
C6—C1—C10	112.58 (13)	H4A—C4—H4B	108.00
C2—C1—C6	112.73 (13)	C4—C5—H5A	109.00
C1—C2—C7	115.59 (15)	C4—C5—H5B	109.00
C3—C2—C7	123.07 (16)	C6—C5—H5A	109.00
C1—C2—C3	121.34 (16)	C6—C5—H5B	109.00
C2—C3—C4	125.20 (16)	H5A—C5—H5B	108.00
C3—C4—C5	113.21 (17)	C2—C7—H7A	109.00
C4—C5—C6	112.80 (15)	C2—C7—H7B	109.00
C1—C6—C5	109.32 (14)	C2—C7—H7C	109.00
C1—C6—C8	110.71 (15)	H7A—C7—H7B	110.00
C5—C6—C8	110.10 (15)	H7A—C7—H7C	110.00
C5—C6—C9	110.39 (15)	H7B—C7—H7C	109.00
C8—C6—C9	109.00 (16)	C6—C8—H8A	109.00
C1—C6—C9	107.28 (15)	C6—C8—H8B	109.00
O1—C10—C1	121.53 (15)	C6—C8—H8C	109.00
O2—C10—C1	115.97 (15)	H8A—C8—H8B	109.00
O1—C10—O2	122.50 (16)	H8A—C8—H8C	109.00
C2—C1—H1	108.3 (9)	H8B—C8—H8C	109.00
C6—C1—H1	108.2 (10)	C6—C9—H9A	109.00
C10—C1—H1	106.1 (9)	C6—C9—H9B	109.00
C2—C3—H3	117.00	C6—C9—H9C	109.00
C4—C3—H3	117.00	H9A—C9—H9B	109.00
C3—C4—H4A	109.00	H9A—C9—H9C	110.00
C3—C4—H4B	109.00	H9B—C9—H9C	109.00
			
C6—C1—C2—C3	−20.0 (2)	C2—C1—C10—O2	120.32 (16)
C6—C1—C2—C7	159.84 (15)	C6—C1—C10—O1	67.1 (2)
C10—C1—C2—C3	105.55 (18)	C6—C1—C10—O2	−114.07 (17)
C10—C1—C2—C7	−74.64 (18)	C1—C2—C3—C4	1.5 (3)
C2—C1—C6—C5	46.66 (18)	C7—C2—C3—C4	−178.26 (18)
C2—C1—C6—C8	168.11 (14)	C2—C3—C4—C5	−11.4 (3)
C2—C1—C6—C9	−73.06 (18)	C3—C4—C5—C6	39.9 (2)
C10—C1—C6—C5	−76.73 (17)	C4—C5—C6—C1	−57.77 (19)
C10—C1—C6—C8	44.72 (19)	C4—C5—C6—C8	−179.60 (17)
C10—C1—C6—C9	163.55 (14)	C4—C5—C6—C9	60.0 (2)
C2—C1—C10—O1	−58.5 (2)		

Symmetry codes: (i) −*x*+1, −*y*+1, −*z*+1; (ii) −*x*+1, *y*−1/2, −*z*+1/2; (iii) −*x*+1, −*y*+1, −*z*; (iv) −*x*+1, *y*+1/2, −*z*+1/2.

### Hydrogen-bond geometry (Å, °)

**Table:**

*D*—H···*A*	*D*—H	H···*A*	*D*···*A*	*D*—H···*A*
O2—H2A···O1^i^	1.01 (4)	1.64 (4)	2.646 (2)	178 (4)
C5—H5A···O1	0.97	2.50	3.133 (2)	122.

Symmetry codes: (i) −*x*+1, −*y*+1, −*z*+1.

## References

[bb1] Bernstein, J., Davis, R. E., Shimoni, L. & Chang, N.-L. (1995). *Angew. Chem. Int. Ed. Engl.* **34**, 1555–1573.

[bb2] Bruker (2008). *APEX2*, *SAINT* and *SADABS* Bruker AXS Inc., Madison, Wisconsin, USA.

[bb3] Etter, M. C., MacDonald, J. C. & Bernstein, J. (1990). *Acta Cryst.* B**46**, 256–262. 10.1107/s0108768189012929 2344397

[bb4] Eugster, C. H., Buchecker, R., Tscharner, C., Uhde, G. & Ohloff, G. (1969). *Helv. Chim. Acta*, **52**, 1729–1731.

[bb5] Fehr, C. & Galindo, J. (1986). *Helv. Chim. Acta*, **69**, 228-235.

[bb6] Fehr, C. & Galindo, J. (1995). *Helv. Chim. Acta*, **78**, 539-552.

[bb7] Heather, J. B., Mittal, R. S. D. & Sih, C. J. (1976). *J. Am. Chem. Soc.* **98**, 3661–3669.

[bb8] Naef, F. & Decorzant, R. (1986). *Tetrahedron*, **42**, 3245-3250.

[bb9] Sheldrick, G. M. (2008). *Acta Cryst.* A**64**, 112–122. 10.1107/S0108767307043930 18156677

[bb10] Snowden, R. L., Muller, B. L. & Schulte-Elte, K. H. (1982). *Tetrahedron Lett.* **23**, 335–338.

[bb11] Spek, A. L. (2009). *Acta Cryst.* D**65**, 148–155. 10.1107/S090744490804362X PMC2631630 19171970

